# Measuring eosinophiluria, urinary eosinophil cationic protein and urinary interleukin-5 in patients with Lupus Nephritis

**DOI:** 10.1186/s13223-014-0061-x

**Published:** 2014-12-12

**Authors:** Tereza Neuma Souza Brito, Maria José Vilar, José Bruno Almeida, Ana Luiza Souza Brito Faria, Sarah Dantas Viana Medeiros, Maria Carmo Cardoso Medeiros, Edna Marques Araújo Silva, Vanessa Marques Araújo Silva, Luanda Bárbara F Canário Souza, Luisa Karla P Arruda, Tatiana Xavier Costa, Geraldo Barroso Cavalcanti Junior, Antonio G Oliveira, Valéria Soraya Farias Sales

**Affiliations:** Department of Clinical and Toxicological Analysis, Postgraduate Program in Health Sciences, Federal University of Rio Grande do Norte, Natal, RN Brazil; Division of Rheumatology, Department of Clinical Medicine, Postgraduate Program in Health Sciences, Federal University of Rio Grande do Norte, Natal, RN Brazil; Division of Nephrology, Department of Integrated Medicine, Federal University of Rio Grande do Norte, Natal, RN Brazil; Clinical Hospital, University of São Paulo, Ribeirão Preto, SP Brazil; Postgraduate Program in Health Sciences, Federal University of Rio Grande do Norte, Natal, RN Brazil; Hospital Universitario Onofre Lopes, Federal University of Rio Grande do Norte, Natal, RN Brazil; Department of Clinical and Toxicological Analysis, Postgraduate in Pharmaceutical Sciences, Federal University of Rio Grande do Norte, Natal, RN Brazil; Department of Clinical Medicine, Faculty of Medicine of Ribeirão Preto, University of São Paulo (FMRP-USP), Ribeirão Preto, SP Brazil; Department of Pharmacy, Postgraduate Program in Health Sciences, Federal University of Rio Grande do Norte, Natal, RN Brazil

**Keywords:** Eosinophils, Systemic lupus erythematosus, Eosinophil cationic protein, Interleukin-5

## Abstract

**Background:**

Urine is increasingly becoming an attractive biological fluid in clinical practice due to being an easily obtained, non-invasive sampling method, containing proteins and peptides. The aim of this study was to investigate eosinophiluria, urinary eosinophil cationic protein (uECP) and urinary IL-5 (uIL-5) in patients with Lupus Nephritis.

**Methods:**

Seventy-four patients with SLE—20 with clinical and laboratory evidence of lupus nephritis (LN group) and 54 without evidence of renal involvement (non-LN group)—were analyzed regarding eosinophiluria, uECP and uIL-5. Eosinophiluria was observed by Hansel's stain, ECP by fluoroenzymeimmunoassay and uIL-5 by quantitative sandwich enzyme immunoassay. Both uECP and urinary IL-5 (uIL-5) were corrected by urinary creatinine. Eosinophiluria and uECP were compared with glomerular erythrocyturia, protein/creatinine ratio (Pr/Cr ratio), serum creatinine, estimated glomerular filtration rate (eGFR), anti-double-stranded DNA (anti-dsDNA), serum levels of complement (C3 and C4), uIL-5/Cr ratio, and SLE disease activity index.

**Results:**

Patients of the LN group had higher eosinophiluria, uECP, uECP/Cr ratio levels, and uIL-5 than patients of the non-LN group (p<0.001 for all). These variables showed a statistically significant correlation with glomerular erythrocyturia, casts, Pr/Cr ratio, serum creatinine, eGFR, anti-dsDNA, uIL-5/Cr, and SLE disease activity index (all p<0.05).

**Conclusion:**

These results provide evidence of increased urinary eosinophils, ECP and IL-5 in patients with SLE and LN; uECP/Cr ratio showed better correlation with markers of renal function and SLE disease activity.

## Background

Systemic lupus erythematosus (SLE) is a multifactorial autoimmune disorder characterized by autoantibody production, immune complex formation, and immunologically mediated tissue injury [1]. Lupus nephritis (LN) is one of the most serious manifestations of SLE, which affects 25–60% of patients, and one of the leading causes of morbidity and mortality of this disease [2-6].

Although the precise pathogenesis of LN has not been fully elucidated, it is mostly attributable to the glomerular deposition of immune complexes and imbalance of the cytokine homeostasis, [7] which leads to a cascade of inflammatory events with recruitment of mononuclear cells, such as T cells, macrophages, and dendritic cells [8]. There is considerable evidence of the role of Th1, Th17, and regulatory T (Treg) cells in SLE, and studies have suggested a possible contribution of Th2 cells [9].

Among the cells that can secrete cytokines capable of promoting T-cell proliferation, activation of Th1, or Th2 polarization is the eosinophil. This is a granulocyte that has been implicated in the modulation of both innate and adaptive immune responses. In response to the diverse stimuli, eosinophils are recruited from the circulation to inflammatory foci where they modulate immune responses through an array of mechanisms, such as secretion of cationic proteins and expression of receptors for cytokines, immunoglobulins, complement, and mRNA for a number of Toll-like receptors. They can initiate antigen-specific immune responses by acting as antigen-presenting cells [10-14].

Once attracted to the site of inflammation, eosinophils becomes activated and four highly cytotoxic cationic protein preformed granules are secreted. They are the eosinophil cationic protein (ECP), eosinophil peroxidase (EPO), eosinophil derived neurotoxin (EDN)/former eosinophil protein X (EPX) and major basic protein (MBP), in addition to chemokines, cytokines, and growth factors. ECP is the best known of these, has been assessed and used as a marker in asthma and other inflammatory diseases, and has been scrutinized in a number of functional studies. Regarding cytokines, IL-5 is the most specific to the eosinophil lineage and is responsible for selective differentiation, regulating growth, activation, and survival of eosinophils [10,15,16].

Based on the findings of eosinophils in the urine of SLE patients and on the role of eosinophils in various inflammatory diseases, this study was aimed to evaluate eosinophiluria, uECP and uIL-5 levels of SLE patients as a possible urinary marker to renal inflammation of SLE patients.

## Patients and methods

### Study population

Patients with SLE diagnosis according to the American College of Rheumatology (ACR) [1] criteria, age ≥18 years, were selected in the Rheumatology Unit Hospital Onofre Lopes (HUOL), Federal University of Rio Grande do Norte (UFRN), Natal, Brazil, a region with high incidence of this disease [17]. Informed consent was obtained from the patients after approval by the local ethics committee, number 044/2006. The study was conducted according to the ethical guidelines of our institution (UFRN) and the Declaration of Helsinki. Disease activity was assessed by the Mexican version of the SLE Disease Activity Index (MEX-SLEDAI) [18]. Patients with immunodeficiency, history of allergy, manifestations of vasculitis, other autoimmune diseases, helminthiasis, prostate cancer, renal cancer, bladder infections, nephrolithiasis, and urinary tract infections were excluded from the study.

The patients were divided into two groups. The first group (the LN group) consisted of patients with active renal manifestations of SLE and clinical and laboratory evidence suggestive of lupus glomerulonephritis. The presence of renal disease activity was defined by MEX-SLEDAI score ≥6 and by the presence of all of the following in the urine test: haematuria ≥1+, proteinuria ≥2+, active urinary sediment with erythrocyturia defined as ≥5 cells/high power field (HPF or 40× magnification), casts (erythrocyte and/or granular, fatty, waxy, and renal tubular epithelial cells), and glomerular dysmorphic erythrocytes. In addition, the patients of the LN group also had to present serum creatinina ≥1mg/dL, eGFR ≤60 mL/min, proteinuria 24-hours ≥3g/L, protein in the first morning urine (spot urine) corrected by creatinine (Pr/Cr ratio) ≥3, positive titers of anti-dsDNA (≥1:40), and decreased concentrations of serum C3 and C4. The second group (the non-LN group) consisted of patients with MEX-SLEDAI score <6 and without laboratory evidence of renal involvement.

All patients were medicated with prednisone at a maximum dose of 10 mg/day and antimalarial drugs. In order to prevent further renal damage, patients in the LN group had prednisone increased to ≥1 mg/kg/day, up to a maximum of one week before inclusion into the study. Once data had been collected, cyclophosphamide 0.5-1.0 g/cm^2^ was added to the prednisone regime, as well as antimalarial drugs, calcium channel blockers, angiotensin-converting enzyme (ACE) inhibitors, proton pump inhibitors, and diuretics.

### Laboratory measurement

The laboratory evaluation included stool analysis, 10 mL of venous blood and, the first morning urine. The venous blood was allowed to clot for 60 min at 24°C, followed by centrifugation (10 min, 24°C, 1600 G). The resulting serum samples were tested for anti-dsDNA, creatinine, eGFR, C3, and C4.

Standard urinalysis for glomerular dysmorphic erythrocytes, eosinophiluria, protein, and creatinine was performed in the first morning urine, midstream. The supernatant (after centrifugation 5 min, 24°C, 400 G) was stored at −80°C until analysis of uECP and uIL-5. Next, this sample was measured and centrifuged (5 min, 24°C, 400 G) for determination of proteinuria and glomerular filtration rate (creatinine clearance). Urinalysis was performed by experienced personnel following the good quality control procedures.

In this study, proteinuria was defined as Pr/Cr ratio, due to a strong correlation between the results found by two methods and in accordance with the recommendation of the Renal Disease Subcommittee of the American College of Rheumatology Ad Hoc Committee on Systemic Lupus Erythematosus Response Criteria [19].

Eosinophiluria assessed by Hansel's stain was conducted after concentrating 50 μL of urine sediment in a cytospin cytocentrifuge. The eosinophils were counted per 10 high-power fields (HPF) and the finding of even a single one was considered positive eosinophiluria [20]. Importantly, this method is analyst-dependent and was therefore evaluated by three experienced analysts.

The uECP measurements were performed by the Pharmacia CAP System® ECP FEIA (fluoroenzymeimmunoassay) (Pharmacia, Uppsala, Sweden), with a coefficient of variation of 2.5%, according to the manufacturer’s instructions. The test is designed as a sandwich immunoassay. Inter- and intra-assay coefficients of variation were less than 8% and the detection limit was 0.5 μg/L. The uECP was determined following the same instructions used for serum ECP, and uECP levels were corrected to urine creatinine (uECP/Cr ratio) with results expressed as micrograms per milligram of creatinine (μg/mgCr).

The concentrations of uIL-5 were determined by quantitative sandwich enzyme immunoassay (Quantikine® Minneapolis, United States of America), according to the manufacturer’s instructions. This immunoassay is a solid phase ELISA designed to measure IL-5 levels in cell culture supernates, serum, plasma, and urine; the detection limit was <3,0 pg/mL. Concentrations of uIL-5 levels were corrected to urine creatinine (uIL-5/Cr ratio) and results were expressed as picrograms per milligram of creatinine (pg/mgCr).

### Statistical analyses

Statistical analyses were performed with SPSS v.17 for Windows (SPSS, Chicago, IL, USA). Continuous variables were tested for normal distribution using the Shapiro-Wilk and Kolmogorov-Smirnov statistical tests. Results are presented as mean±standard deviation (SD), median, interquartile range (IQR), and minimum (min) and maximum (max) values. uECP correlation with disease activity and renal function tests was assessed using Pearson and Spearman's rank correlation coefficient.

Categorical variables were compared between groups with the chi-square test and continuous variables were compared with the Student's t-test and the Mann-Whitney’s U-test. Two-tailed *p-values* less than 0.05 were regarded as statistically significant. Assuming a significance level (α) of 0.05 and a power (1 – β) of 80%, the sample size used was able to detect statistical significance for differences between groups of 1.5 cells in eosinophiluria, 3.9 μg/L in uECP, and 6.5 μg/mgCr in uECP/Cr ratio.

## Results

### Baseline characteristics of patients

A total of 74 patients with SLE—20 patients (16 women and 4 men) with active SLE and evidence of LN (MEX-SLEDAI score 5.0-22.0) and 54 patients (all women) with inactive disease and without LN (MEX-SLEDAI score <1)—were evaluated for eosinophiluria, uECP, and uIL-5. The demographic characteristics and laboratory parameters of the two groups are presented in Table 1. The mean age was 29.5±8.5 years and the mean disease duration was 67.0±58.5 months (range 1–228 months). There were no significant differences in ethnicity between the two groups of patients. Only three patients of the LN group also received one intravenous pulse of cyclophosphamide (0.5 g/m^2^ body surface area) in the same period.

**Table 1:** **Patient characteristics and laboratory parameters in SLE patients with and without lupus nephritis (LN)**

	**LN group (n=20)**	**non-LN group (n=54)**
**Age (y) mean±SD**	26.6±5.1*	30.5±9.3
**Gender (men/women) %**	20/80*	0/100
**Caucasian (W/N-W) %**	30/70	33/67
**Disease duration of SLE (months) mean±SD**	30±26.3*	80.8±61.3
**Mex-SLEDAI mean±SD**	9.20±4.57*	0.07±0.38
**Dipstick positive hematuria - (1+ to 4+), % (n)**	100.0 (20)*	0.0 (0)
**Dysmorphic erythrocyte, % (n)**	70.0 (14)*	1.8 (1)
**Erythrocyte, granular, fatty and waxy casts, % (n)**	70.0 (14)*	7.4 (4)
**Oval fat bodies, % (n)**	80.0 (16)*	1.8 (1)
**Erythrocytes sedimentation rate/HPF mean±SD**	33.8±16.3*	1.9±1.7
**Dipstick positive proteinuria (1+ to 4+), % (n)**	100.0 (20)*	5.6 (3)
**Pr/Cr ratio (mg/mg) mean±SD**	4.89±4.30*	0.37±0.26
**24-h Proteinuria (g/L) mean±SD**	4.77±3.58*	0.23±0.20
**Serum creatinine (μmol/L) mean±SD**	140.42±73.70*	70.90±16.92
**eGFR (mL/min) mean±SD**	57.27±28.02*	95.22±23.94
**Creatinine clearance (mL/min/1.73 m** ^**2**^ **) mean±SD**	60.33±24.99*	92.68±22.97
**Serum C3 (mg/L) mean±SD**	62.83±42.40*	131.30±64.24
**Serum C4 (mg/L) mean±SD**	27.87±14.92*	44.48±18.38
**Anti-dsDNA title mean±SD**	98.95±50.54*	17.22±9.20

HPF, high power field or 400X.

*p-value <0.05.

### Laboratory findings

**Figure 1:**
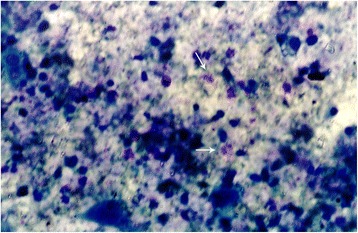
**Eosinophiluria by Hansel’s stain (400X) (arrow).**

**Figure 2:**
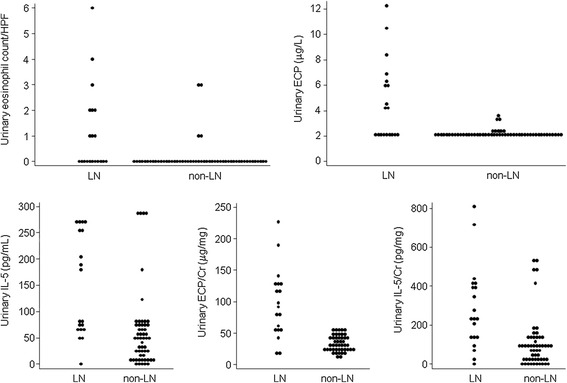
**Urinary eosinophil count/HPF, ECP (μg/L), IL-5 (pg/mL), ECP/Cr (μg/mg) and IL-5/Cr (pg/mg) in SLE patients with and without lupus nephritis (LN).**

Eosinophiluria (as shown in Figure 1) was observed in 45% (n=9) of the patients in the LN group and 5.6% (n=3) in the non-LN group. The mean urinary eosinophil count /HPF was significantly higher in patients in the LN group than that in patients in the non-LN group (*p*<0.001). The concentrations of uECP, uECP/Cr ratio, uIL-5, and uIL-5/Cr ratio were higher in the LN group than in the non-LN group (*p* <0.05), (Table 2, Figure 2).

**Table 2:** **Laboratory values for 74 SLE subjects with and without renal disease**

	**LN group (n=20)**	**non-LN group (n=54)**	***p-*** **value**
**Eosinophiluria, % (n)**	45.0 (9.0)*	5.6 (3.0)	<0.001
**Urinary eosinophil count /HPF mean±SD**	1.1±1.65*	0.15±0.59	<0.001
**Urinary ECP (μg/L), mean±SD**	4.58±3.18*	2.12±0.36	<0.001
**Urinary ECP/Cr ratio (μg/mgCr) mean±SD**	96.56±53.70*	34.55±12.66	<0.001
**Urinary IL-5 (pg/mL) mean±SD**	144.52±95.59*	61.68±73.51	<0.001
**Urinary IL-5/Cr ratio (pg/mgCr) mean±SD**	299.72±208.63*	106.21±134.898	<0.001

HPF, high power field or 400X; ECP, eosinophil cationic protein; uECP/Cr ratio, eosinophil cationic protein-creatinine ratio; uIL-5, urinary Interleukin-5.

*p-value <0.05.

In addition, a statistically significant correlation was observed between study variables and markers of active renal disease (Table 3), such as haematuria, glomerular dysmorphic erythrocytes, casts, Pr/Cr ratio, serum creatinine, eGFR, anti-dsDNA, serum C3, serum C4, and SLE disease activity index (all p<0.05). The strongest associations were observed between uECP/Cr ratio and haematuria (r_s_=0.76), Pr/Cr ratio (r_s_=0.75), serum creatinine (r_s_=0.70) and MEX-SLEDAI (r_s_=0.72), *p*<0.001. Urinary IL-5 and uIL-5/Cr ratio showed a statistically significant correlation with eosinophiluria, uECP and uECP/Cr ratio (p<0.05). The uIL-5/Cr also showed a statistically significant correlation with MEX-SLEDAI (r_s_=0.41), *p*<0.01.

**Table 3:** **Rank correlation between study variables and laboratory parameters that evaluate the involvement of renal function and disease activity in SLE**

	**Eosinophiluria**	**Urinary ECP**	**uECP/Cr ratio**
**Haematuria**	0.52*	0.49	0.76*
**Glomerular dysmorphic erythrocyte**	0.51*	0.56*	0.52*
**Casts**	0.44	0.45	0.63*
**Pr/Cr ratio**	0.46	0.55*	0.75*
**Serum creatinine**	0.59*	0.57*	0.70*
**eGFR**	−0.54*	−0,47	−0.61*
**Anti-dsDNA**	0.26	0.27	0.58*
**Serum C3**	−0.21	−0.16	−0.30
**Serum C4**	−0.28	−0.44	−0.31
**Urinary IL-5**	0.31	0.32	0.27
**Urinary IL-5/Cr (pg/mgCr)**	0.36	0.28	0.50*
**Mex-SLEDAI**	0.41	0.44	0.72*

ECP, eosinophil cationic protein; Pr/Cr ratio, protein-creatinine ratio; C3, complement 3; C4, complement 4; Mex-SLEDAI, Mexican version of the SLE Disease Activity Index. *p-value <0.05.

## Discussion

This study is the first to investigate eosinophiluria, uECP and uIL-5 as a possible marker to evaluate renal inflammatory activity of SLE patients. We selected patients with clinical and laboratory features strongly suggestive of LN, and control patients without evidence of LN. This study revealed a statistically significant increase in eosinophiluria, uECP and uIL-5 in patients with LN compared with patients without LN.

Lupus nephritis requires long-term monitoring over several years, as flares as well as progressive deterioration of renal function may occur. Evaluation for LN includes dipstick and urine sediment analysis, urinary protein and creatinine excretion, determination of serum creatinine and assessment of serological markers such as anti-dsDNA antibody titres and C3 and C4 levels. The combination of these markers is a powerful measure for the detection of active renal manifestations of SLE. However renal biopsy remains the gold standard to assess disease severity, but multiple biopsies to gauge treatment efficacy are not feasible due to their invasive nature with risks of bleeding and infection, thereby presenting a less satisfactory method for monitoring renal involvement in SLE [21-23].

Urinary biomarkers may also reflect to some extent the degree of tubular dysfunction, rather than purely reflecting underlying glomerular pathology [24]. A biomarker that could forecast lupus nephritis flares well before thresholds of proteinuria, renal function and urine sediment that signal clinical flare are reached would be a valuable tool [25]. Thus, novel biomarkers that are able to discriminate lupus renal activity and its severity, predict renal flares, and monitor treatment response and disease progress are clearly necessary [26,27].

The clinical and laboratory differences presented between the two groups of patients selected for this study were consistent with literature data. Marks et al. [28] found that LN patients had higher urine albumin/creatinine ratios compared to non-nephritis patients. Rubinstein et al. [29] found proteinuria (Pr/Cr ratio) greater than 2.0, decreased creatinine clearance, and SLE disease activity index (SLEDAI) ≥4 in SLE patients with biopsy-proven nephritis. A study by Guo et al. [30] found, in patients with LN class IV, values of 24-h proteinuria ≥3 g/day, increased serum creatinine, and a mean SLEDAI ≥9 in all patients, suggesting that most SLE patients with renal diseases were in the active stage.

Urine as a biological sample has the advantage that it is easily obtainable by non-invasive means and thus allows investigators to avoid many regulatory hurdles [31]. This study showed that eosinophiluria, uECP, uECP/Cr and uIL-5 levels were higher in the LN group than in the non-LN group. Eosinophiluria has a higher frequency in the LN group and showed a consistent and statistically significant correlation with a number of evaluated parameters of renal inflammation and dysfunction. Eosinophiluria has been suggested to be useful in establishing the diagnosis of acute interstitial nephritis. Some diseases of the urinary tract are accompanied by pyuria and eosinophilic tissue infiltration but have not been tested for urinary eosinophils. These diseases include eosinophilic prostatitis, eosinophilic ureteritis, eosinophilic infiltration surrounding bladder cancer, eosinophilic glomerulonephritis, and atheroembolic renal disease [32-34].

As renal biopsies were not performed in our study, we are not able to unequivocally state that the positive eosinophiluria was entirely of renal origin. As a matter of fact, one might question whether that eosinophiluria could be due to active disease rather than to lupus nephritis. However, to classify the two study groups based on renal biopsy would require that a large number of patients with no clinical or laboratorial evidence of renal lesion had to be needlessly submitted to the risk of renal biopsy. Therefore, in order to avoid exposing study subjets to that risk at the same time to minimize the chances that patients without lupus nephritis could be included in the LN group, we imposed the constraint that patients were classified in the LN group only if they had active SLE, in addition to clinical and laboratorial manifestations of renal disease. Conversely, the chances of including patient with lupus nephritis in the control group were minimized by excluding subjects with manifestations of renal disease or active SLE. Although this design caused nephritis to be statistically confounded with active disease, we are convinced that eosinophiluria is not related to active SLE, a conviction that is further reinforced by the observation that there was no statistically significant difference (p=0.52) in the absolute count of eosinophils in the peripheral blood between the LN group (142.7±32.5/ml) and controls (160.3±20.4).

The literature does not report the participation of eosinophils in the inflammatory process of LN. However, studies probing the immunobiology of eosinophils have uncovered an evolving story in which eosinophils participate in innate immune processes as more than terminal effector cells. An appreciation of chemokines, cytokines and growth factors derived from their granules, both in circulation and in tissues, provides the basis for many recently discovered functions of that cell, including regulation of the immune microenvironment, inflammatory response, homeostasis and tissue remodeling. The demonstration that eosinophils can be antigen-presenting cells in mice and humans, establish eosinophils as cells that participate in innate and adaptive immunity [10,12,35]. Thus, based on these data we can suggest that eosinophils may play a role in kidney inflammation in LN.

Studies have shown that during inflammation whole eosinophil granules are released from disrupted cells and those internal proteins are subsequently released differentially through the process of piecemeal degranulation. Among the components of these granules, ECP is RNAse A superfamily, protein rich in arginine residues, which gives a high concentration of positive charges, promoting a strong attraction for molecules negatively charged existing in cell membranes [15,16,36]. This property may explain its cytotoxic power in the cell membranes causing the formation of pores or channels on the surface of the membrane, disrupting its lipid structure and possibly facilitating the entry of other cytotoxic molecules. Sensitive assays have been developed for its measurement in biological fluids which have contributed to the understanding of the role of the eosinophils in disease [10,15].

This study demonstrated increased uECP in patients of the LN group compared to non-LN group patients and a statistically significant correlation between the concentration of uECP and haematuria, glomerular dysmorphic erythrocytes, casts, Pr/Cr ratio, serum creatinine, eGFR, uIL-5, and MEX-SLEDAI. Interestingly, after correction of uECP by creatinine (ECP/Cr ratio), an increase was noted in the correlation with haematuria, Pr/Cr ratio, serum creatinine and MEX-SLEDAI.

Inflammatory diseases tend to share common pathways and thus many a potential biomarker will not be specific for a particular disease. Few biomarkers for SLE have been validated and employed for making clinical decisions given the complex etiopathogenesis, heterogeneous clinical manifestations, and varying rates of disease progression among individual SLE patients [3,27].

In the present study, uIL-5 had higher concentration in the LN group and after being corrected by creatinine (uIL-5/Cr ratio) showed better correlation with the uECP/Cr ratio and with MEX-SLEDAI. No article has yet been published in the literature about the uIL-5/Cr ratio in SLE. The increased levels of uIL-5 can justify the appearance of eosinophils and ECP in urine of the SLE patients studied because the cytokine IL-5, which is a key Th2 cytokine in eosinophil biology, is involved in eosinophil differentiation, maturation, migration and activation of these cells [37].

This is the first study showing that eosinophiluria, urinary ECP and IL-5 may be useful as biomarkers of renal inflammation in SLE patients. The data are encouraging and provide a basis for future research. Our findings not only suggest that uECP/Cr ratio may be a urinary biomarker of renal inflammatory activity in SLE patients but also shows the need to investigate the role of eosinophils in the inflammatory process of nephritis in patients with systemic lupus erythematosus.

In conclusion, we showed increased urinary eosinophils, uECP/Cr ratio and uIL-5 levels in patients with history of lupus nephritis. Urinary ECP/Cr ratio might serve as a novel marker of renal inflammation in SLE. Evidently, more research is needed to verify the behavior of these biomarkers in both groups.

## References

[1] Hochberg MC (1997). Updating the American College of Rheumatology revised criteria for the classification of systemic lupus erythematosus. Arthritis Rheum.

[2] Gordon C, Jayne D, Pusey C, Adu D, Amoura Z, Aringer M, Ballerin J, Cervera R, Calvo-Alén J, Chizzolini C, Dayer JM, Doria A, Ferrario F, Floege J, Guillevin L, Haubitz M, Hiepe F, Houssiau F, Lesavre P, Lightstone L, Meroni PL, Meyer O, Moulin B, O’Reilly K, Praga M, Schulze-Koops H, Sinico RA, Smith KGC, Tincani A, Vasconcelos C, Hughes G (2009). European consensus statement on the terminology used in the management of lupus glomerulonephritis. Lupus.

[3] Liu CC, Ahearn JM (2009). The search for lupus biomarkers. Best Pract Res Clin Rheumatol.

[4] Borchers AT, Naguwa SM, Shoenfeld Y, Gershwin ME (2010). The geoepidemiology of systemic lupus erythematosus. Autoimmun Rev.

[5] Lightstone L (2010). Lupus nephritis: where are we now?. Curr Opin Rheumatol.

[6] Valesini G, Conti F (2011). The Persistent Challenge of Lupus Nephritis. Clin Rev Allergy Immunol.

[7] Dolff S, Abdulahad WH, van Dijk MCRF, Limburg PC, Kallenberg CGM, Bijl M (2010). Urinary T cells in active lupus nephritis show an effector memory phenotype. Ann Rheum Dis.

[8] Tucci M, Stucci S, Strippoli S, Silvestris F (2010). Cytokine overproduction, T-cell activation, and defective T-regulatory functions promote nephritis in systemic lupus erythematosus. J Biomed Biotechnol.

[9] Charles N, Hardwick D, Daugas E, IIIei GG, Rivera J (2010). Basophils and the T helper 2 environment can promote the development of lupus nephritis. Nat Med.

[10] Rothenberg ME, Hogan SP (2006). The eosinophil. Annu Rev Immunol.

[11] Jacobsen EA, Taranova AG, Lee NA, Lee JJ, Ariz S (2007). Eosinophils: Singularly destructive effector cells or purveyors of immunoregulation?. J Allergy Clin Immunol.

[12] Hogan SP, Rosenberg HF, Moqbel R, Phipps S, Foster PS, Lacy P, Kay AB, Rothenberg ME (2008). Eosinophils: biological properties and role in health and disease. Clinical Exp Allergy.

[13] Woschnagg C, Rubin J, Venge P (2009). Eosinophil cationic protein (ECP) is processed during secretion. J Immunol.

[14] Kampe M, Stolt I, Lampinen M, Janson C, Stalenheim G, Carlson M (2011). Patients with allergic rhinitis and allergic asthma share the same pattern of eosinophil and neutrophil degranulation after allergen challenge. Clin Mol Allergy.

[15] Bystrom J, Amin K, Bishop-Bailey D (2011). Analysing the eosinophil cationic protein - a clue to the function of the eosinophil granulocyte. Respir Res.

[16] Acharya RK, Ackerman SJ (2014). Eosinophil Granule Proteins: Form and Function. J Biol Chem.

[17] Vilar MJP, Sato EI (2002). Estimating the incidence of systemic lupus erythematosus in a tropical region (Natal, Brazil). Lupus.

[18] Bombardier C, Gladman DD, Urowitz MB, Caron D, Chang CH (1992). Derivation of the sledai. A disease activity index for lupus patients. Arthritis Rheumatism.

[19] Liang MH, Schur PH, Fortin P, St Clair W, Balow JE, Costenbader K, Crofford L, de Pablo P, Dooley MA, Finckh A, Gordon CP, Lund-berg IE, Meyrier A, Nived O, Ponticelli C, Schneider MK, Singh A, Wallace DJ (2006). The American college of rheumatology response criteria for proliferative and membranous renal disease in systemic lupus erythematosus clinical trials. Arthritis Rheumatism.

[20] Nolan CR, Anger MS, Kelleher SP (1986). Eosinophiluria–a new method of detection and definition of the clinical spectrum. N Engl J Med.

[21] Christopher-Stine L, Siedner M, Lin J, Haas M, Parekh H, Petri M, Fine DM (2007). Renal biopsy in lupus patients with low levels of proteinuria. J Rheumatol.

[22] Kon T, Yamaji K, Sugimoto K, Ogasawara M, Kenpe K, Ogasawara H, Yang KS, Tsuda H, Matsumoto T, Hashimoto H, Takasaki Y (2010). Investigation of pathological and clinical features of lupus nephritis in 73 autopsied cases with systemic lupus erythematosus. Mod Rheumatol.

[23] Dolff S, Abdulahad WH, Arends S, van Dijk MCRF, Limbrug PC, Kallenberg CGM, Bijl M (2013). Urinary CD8+ T-cell counts discriminate between active and inactive lupus nephritis. Arthritis Res Therapy.

[24] Adhya Z, Borozdenkova S, Karim MY (2011). The role of cytokines as biomarkers in systemic lupus erythematosus and lupus nephritis. Nephrol Dial Transplant.

[25] Rovin BH, Zhang X (2009). Biomarkers for lupus nephritis: the quest continues. Clin J Am Soc Nephrol.

[26] Manoharan A, Madaio MP (2010). Biomarkers in lupus nephritis. Rheum Dis Clin North Am.

[27] Mok CC (2010). Biomarkers for lupus nephritis: a critical appraisal. J Biomed Biotechnol.

[28] Marks SD, Shah V, Pilkington C, Tullus K (2010). Urinary monocyte chemoattractant protein-1 correlates with disease activity in lupus nephritis. Pediatr Nephrol.

[29] Rubinstein T, Pitashny M, Levine B, Schwartz N, Schwartzman J, Weinstein E, Pego-Reigosa JM, Lu TY, Isenberg D, Rahman A, Putterman C (2010). Urinary neutrophil gelatinase-associated lipocalin as a novel biomarker for disease activity in lupus nephritis. Rheumatology (Oxford).

[30] Guo Q, Lu X, Miao L, Wu M, Lu S, Luo P (2010). Analysis of clinical manifestations and pathology of lupus nephritis: a retrospective review of 82 cases. Clin Rheumatol.

[31] Li Y, Tucci M, Narain S, Barnes EV, Sobel ES, Segal MS, Richards HB (2006). Urinary biomarkers in lupus nephritis. Autoimmun Rev.

[32] Corwin HL, Bray RA, Haber MH (1989). The detection and interpretation of urinary eosinophils. Arch Pathol Lab Med.

[33] Ruffing KA, Hoppes P, Blend D, Jarjoura D, Whittier FC (1994). Eosinophils in urine revisited. Clin Nephrol.

[34] Sutton JM (1986). Urinary eosinophils. Arch Intern Med.

[35] Shamri R, Xenakis J, Spencer L (2011). Eosinophils in innate immunity: an evolving story. Cell Tissue Res.

[36] Mallorquí-Fernández G, Pous J, Peracaula R, Aymamí J, Maeda T, Tada H, Yamada H, Seno M, de Llorens R, Gomis-Rüth FX, Coll M (2000). Three-dimensional crystal structure of human eosinophil cationic protein (RNase 3) at 1.75 Å resolution. J Mol Biol.

[37] Kim CK, Kita H, Callaway Z, Kim HB, Choi J, Fujisawa T, Shin BM, Koh YY (2010). The roles of a Th2 cytokine and CC chemokine in children with stable asthma: Potential implication in eosinophil degranulation. Pediatr Allergy Immunol.

